# Cyclopedia of the Diseases of Children

**Published:** 1891-01

**Authors:** 


Cyclopedia of the Diseases of Children, Medical and Surgical. The articles written especially for the work by American, British and Canadian authors. Edited by John M. Keating, M. D. Vol. IV., illustrated. Philadelphia : J. B. Lippincott Company. 1890.

The fourth volume of this magnificent work is on a par with the preceding volumes, in the excellence of its articles. The subjects treated of are the Eye, the Ear, General Hygiene, and Diseases of the Nervous System. The whole volume is profusely and beautifully illustrated. The articles on hygiene include physical development, massage, prophylaxis of disease in children, school-hygiene, construction of children's hospitals, juvenile crime and public methods of prevention and reclamation, and medico-legal testimony. The first and fourth of these are especially worthy of commendation. The diseases of the nervous system are fully and satisfactorily treated of in a series of elaborate articles by observers who have made a special study of such cases. Especially worthy of note is the article on Diagnosis of Nervous Diseases by Allan McLane Hamilton. The letter-press is in every way most satisfactory.

DeL. R.

